# Hemispheric Lateralization in Older Adults Who Habitually Play Darts: A Cross-Sectional Study Using Functional Near-Infrared Spectroscopy

**DOI:** 10.3390/healthcare12070734

**Published:** 2024-03-27

**Authors:** Koki Toyofuku, Satoru Hiwa, Kensuke Tanioka, Tomoyuki Hiroyasu, Masaki Takeda

**Affiliations:** 1 Graduate School of Life and Medical Sciences, Doshisha University, Kyoto 610-0394, Japan; 2 Department of Biomedical Sciences and Informatics, Doshisha University, Kyoto 610-0394, Japan; ktanioka@mail.doshisha.ac.jp (K.T.); tomo@is.doshisha.ac.jp (T.H.); 3 Faculty of Health and Sports Science, Doshisha University, Kyoto 610-0394, Japan; mtakeda@mail.doshisha.ac.jp

**Keywords:** exercise training, darts, functional near-infrared spectroscopy, cognitive function, hemispheric lateralization

## Abstract

Exercise training integrating physical and cognitive activities is gaining attention because of its potential benefits for brain health. This study focuses on exercise training using a dart game called Wellness Darts. Wellness Darts is a sport involving throwing darts and walking to pull them out of the board, memorizing the score, and subtracting this from the total score, thus requiring the simultaneous performance of two tasks: exercise and calculation. This is expected to maintain and improve cognitive function, and whether this continual darts training affects brain function is of great interest. Before conducting the longitudinal study revealing its effect on brain function, we aimed to cross-sectionally confirm the difference in hemispheric lateralization between expert and non-expert players. Functional near-infrared spectroscopy (fNIRS) was used to measure brain activity for three groups: an expert older group who practiced darts continually, a non-expert older control group, and a non-expert younger control group. Their brain activity patterns were quantified by the lateralization index (LI) and compared between groups. The results showed that the younger and the expert older groups had significantly higher LI values than the non-expert older group, and there was no difference between the expert older and the younger groups. Our results suggest that the Wellness Darts game possibly promotes hemispheric lateralization.

## 1. Introduction

Cognitive decline is one of the most crucial issues to be addressed in the aging population. Various interventions have been proposed to maintain and improve cognitive function in older adults, including physical activities and cognitive exercises [1,2,3]. In this context, exercise and sports that integrate physical and cognitive activities are gaining attention because of their potential benefits for brain health.

Recent studies highlight the benefits of exercise for cognitive function in older adults, emphasizing the importance of integrating physical and cognitive activities for enhancing brain health. Previous studies have shown that mind–body exercises like tai chi and dance [4] and moderate aerobic activities can improve cognitive functions such as global cognition, cognitive flexibility, and working memory [5]. Furthermore, several studies have suggested that combined physical and cognitive activity programs effectively prevented cognitive decline [6], with interactive training showing significant improvements in cognitive and physical functions [7]. These findings underscore the potential of exercise training, particularly programs that combine physical and cognitive activities, as an effective intervention for maintaining and improving brain health [8,9].

Inspired by the results of these studies, this study explores how exercise training, particularly with the darts game “Wellness Darts”, is expected to affect brain and cognitive function in older adults. Darts is one of the sports that provides fun and gameplay; players must subtract the score obtained from the points they have and reduce the total score to zero. Wellness Darts also has the unique feature of involving rote score calculations [10]. “Rote”, in this case, refers to the process of quickly mentally calculating the score of a dart thrown by a player on a dartboard and subtracting it from the points the player has. Wellness Darts is a combined physical and cognitive activity program that involves not only throwing darts, but also walking to pull them out of the board, memorizing the score, and subtracting it from the total score, thus allowing the performance of two tasks at the same time: exercise and calculation [10]. Simultaneous performance of the motor and cognitive tasks, each effective in improving cognitive function as a single task, is more effective in increasing PFC activity and improving cognitive function than performing either task alone [11]. Takeda et al. conducted cognitive tests before, one month, and two months after an experiment on a Wellness Darts intervention group and a control group of older adults [10]. The results showed that continuing Wellness Darts increased older adults’ short-term memory test (STM test) scores. Understanding how Wellness Darts can improve cognitive function is essential for developing interventions that are especially beneficial for older adults with cognitive disorders such as dementia and mild cognitive impairment (MCI). However, the impact of continual practice of Wellness Darts on brain function is still being determined. To develop Wellness Darts as a combined physical and cognitive activity program, it is essential to understand its potential effects on brain function.

Several studies have investigated the association between the cognitive function of older adults and brain function. These investigations are beneficial in determining what measures and which brain regions should be used for the brain analysis in our study. The Hemispheric Asymmetry Reduction in OLDer adults (HAROLD) model proposed by Cabeza et al. asserts that the degree of frontal lobe (FL) lateralization seen in youth decreases with age [12,13,14,15,16,17,18,19]. In addition, Erickson et al. reported that when cognitive training was conducted on healthy older adults, lateralization was observed with improved cognitive performance, bringing the brain activation pattern closer to that of younger adults [15]. That is why increased lateralization can be used as a measure of cognitive improvement. On the other hand, brain regions such as the inferior frontal gyrus (IFG), superior frontal gyrus (SFG), middle frontal gyrus (MFG) [20], and DLPFC have been reported to be activated during mental calculation [21]. Furthermore, the inferior parietal lobule (IPL) in patients with cognitive decline, such as MCI and Alzheimer’s disease, is reported to be less activated [22].

The general objective of this study is to examine the cognitive status and brain function of participants who are involved in the Wellness Darts program. The study cross-sectionally compares the outcomes across three different groups—older adults who are skilled in darts, older adults who are new to darts, and younger adults who have no experience with darts. We have proposed three hypotheses to achieve this goal: (1) older adults who practice Wellness Darts (referred to as the ‘expert older group’) will show a level of hemispheric lateralization similar to that of younger people who have never played Wellness Darts before (referred to as ‘younger group’ as a control), with activation of the dorsal superior frontal gyrus (SFGdor) and MFG, which are known to be activated during mental arithmetic. (2) There is no difference in the level of IPL activation between the expert older group and the younger group. (3) There is a positive correlation between the level of hemispheric lateralization in brain activity and the number of days of Wellness Darts experience in the expert older group. These three hypotheses were tested by measuring brain activity using functional near-infrared spectroscopy (fNIRS), a non-invasive functional brain imaging system that is robust to body movement. The third group, the older adults who had never played Wellness Darts (referred to as the ‘non-expert older group’), was used as the older control to confirm that hypotheses (1) and (2) were valid only for the expert older group.

## 2. Materials and Methods

### 2.1. Participants

There were 21 healthy younger adults (12 males, 9 females; mean age: 22.5 ± 0.8 years) who had never played Wellness Darts, 21 healthy older adults (8 males, 13 females; mean age: 75.1 ± 4.3 years) who continually played Wellness Darts, and 21 healthy older adults (4 males, 17 females; mean age: 74.1 ± 4.5 years) who had never played Wellness Darts participated in the experiment. These three participant groups were referred to as the younger group, the expert older group, and the non-expert older group. The participants for the expert older group were recruited from Wellness Darts players in the Kyotanabe-Doshisha Sports Club (KDSC), one of the community sporting clubs in Kyoto, Japan. The participants for the non-expert group were also recruited based on their acquaintanceship with members of the expert older group. Only healthy participants over 60 could participate in this study for these two groups. For the younger group, the healthy undergraduate and graduate school students aged over 20 were recruited from Doshisha University, Kyoto, Japan. The sample size for each group was determined with a significance level of 0.05 and a power of 0.8 to detect the effect size of Cohen’s *d* = 0.79 reported in a previous study comparing the degree of hemispheric lateralization in older adults using fNIRS [23] (G*Power was used for the calculation).

### 2.2. Wellness Darts Task

Wellness Darts is a darts game developed by the Japan Wellness Darts Association. The standard game of Wellness Darts is the “Zero One Game 251”. In the game, each player is assigned a score of 251 and throws darts until the score reaches 0, subtracting the score of each dart thrown from the holding points. The first player to reach a score of exactly 0 wins. Each player can throw darts three times in their turn (which is defined as one set), and is required to make their score exactly zero on the third throw for either of five sets. In addition, each player must subtract the points earned from their score by themselves. We assumed that these mental arithmetic operations, the planning of throws to reach zero as quickly as possible, and the exercise associated with throwing darts could be effective in improving cognitive function because of the simultaneous performance of motor and cognitive tasks said to be effective in increasing PFC activity and improving cognitive function [11]. However, since the official rules of Wellness Darts place a physical burden on the participants due to the long duration of the experiment, in this study, the initial score of the subjects was set at 128 points, and each subject played the game for a maximum of five sets. Furthermore, the game was terminated even if the score did not reach zero in the fifth set.

### 2.3. Procedure

The participants played up to five sets of Wellness Darts with a maximum of 128 holding points, while brain activity and head acceleration were simultaneously recorded. As described below, the accelerations were used to remove noise due to head movements. In each of the three throws in one set of the Wellness Darts game, the participants were instructed by the recorded voice prompts and the beep cue from the experimental computer on when to hold the dart, throw, and start the calculation; the participants performed each action following the instructions.

During the planning block set at the beginning of each throwing session, participants were asked to determine where to hit (designate scoring points) within 5 s for the first throw and within 13 s for the remaining two throws. At the beginning of the second and third throwing sessions, they were provided an extra 8 s in the planning blocks to tally the points from the previous throws. Of note, we excluded the beginning 8 s. Instead, we applied the later 5 s for the subsequent analyses because we assumed that the first throw did not involve the confirmation of the hit point and employed different cognitive processes than those used in determining where to hit. The planning block was followed by dart-throwing within 3 s after the beep cue.

**Figure 1:**
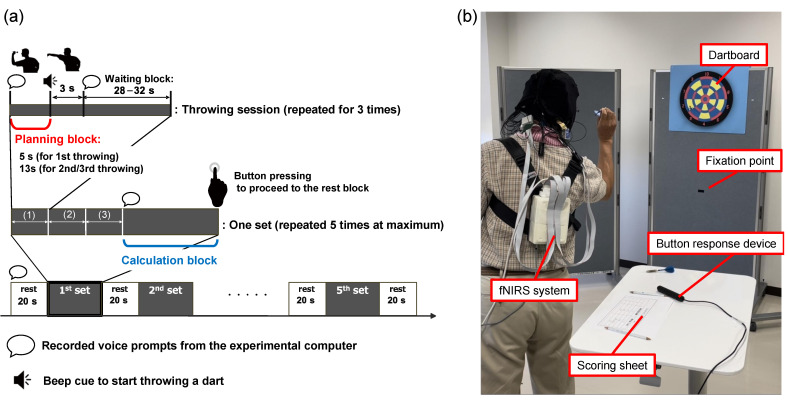
Overview of the experiment. (**a**) The experimental procedure. (**b**) The experimental setup.

Following dart throwing, the participants were instructed to move their gaze away from the dartboard to the fixation point and not to think of anything in particular. This waiting block consisted of a random duration between 28 and 32 s to reduce the anticipatory response. After completing the three throwing sessions, the participants commenced the calculation block, where they were instructed to subtract the scores they received during the previous throws from the total score, and write the remaining points on the scoring sheet. Then, they were asked to press a button to proceed to the 20 s rest block. During the rest block, they stayed the same as in the waiting block. This procedure was repeated for five sets. The overall procedure is illustrated in Figure 1.

All participants were informed of the methods and risks of the experiment and signed a written informed consent form. This study was conducted in accordance with the Research Ethics Committee of Doshisha University (Kyoto, Japan) (approval code: 20014). Before the experiment, participants were asked to complete a questionnaire, a dart-throwing performance test, and five cognitive tests: a simple cognitive (SC) test, a computational ability test, a short-term memory (STM) test, a trail-making peg test (TMPT), and CogEvo. Then, the participants performed one set of dart-throwing before being fitted with the fNIRS device to understand the experimental procedure.

### 2.4. fNIRS Data Acquisition

fNIRS is an optical neuroimaging method that assesses cerebral blood flow alterations by emitting near-infrared light onto the scalp and detecting changes in light absorption with a detector located about 30 mm away from the emitter. This absorption fluctuates with variations in oxygenated and deoxygenated hemoglobin concentration; therefore, fNIRS enables observations of hemodynamic responses associated with brain activity. It is non-invasive and requires minimal restrictions during the measurement, so it has advantages for monitoring individuals—such as infants or subjects engaged in physical activity—who are otherwise challenging to assess using other modalities. Given these features, fNIRS is one of the most promising tools in medicine and cognitive neuroscience.

**Figure 2:**
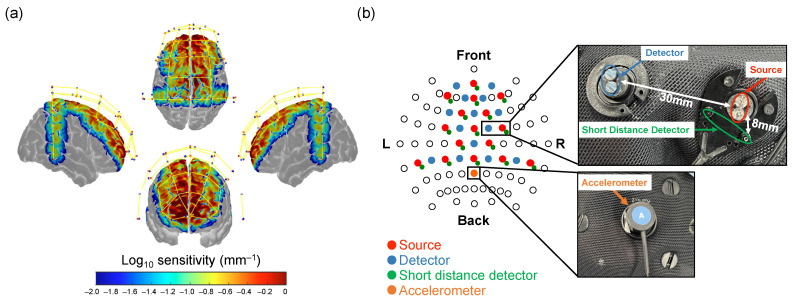
Probe displacement of fNIRS. (**a**) Monte-Carlo simulation results over the frontal cortex, temporal cortex, and parietal cortex. Red dots represent emitters, blue dots represent detectors, and yellow lines represent measurement channels (created using Homer 2 AtlasViewer; v2.8, p2.1: https://www.nitrc.org/frs/shownotes.php?release_id=3956; accessed on 27 March 2024). The color bar represents the spatial sensitivity of the fNIRS measurements. (**b**) The two-dimensional fNIRS montage using the International 10–20 measurement system as reference is presented.

Changes in the concentration of oxyhemoglobin (HbO), deoxyhemoglobin (HbR), and total hemoglobin (HbT) in the participants were measured using NIRSport2 (NIRx Medical Technologies, LLC, Minneapolis, MN, USA). The sampling frequency was 4.36 Hz, and the wavelengths of the near-infrared light were 760 and 850 nm [24]. As shown in Figure 2b, 16 sources and 14 detectors were placed on the frontal, parietal, and temporal regions of the head. A pair of adjacent sources and detectors at 30-millimeter intervals constituted 1 standard channel, and 44 standard channels were placed. In addition, a short-distance detector bundle (NIRx Medical Technologies, LLC, Minneapolis, MN, USA) was established to eliminate extra-cerebral blood flow alterations [25,26]. The signals obtained from the 16 short-distance channels were used to detect extracerebral signals and to remove the effect of skin blood flow in the signals measured by the standard channels. To remove noise from body motion, a six-axis accelerometer (NIRx Medical Technologies, LLC, Minneapolis, MN, USA) was used to measure head accelerations at a sampling frequency of 4.36 Hz, the same frequency as the change in hemoglobin concentration [27]. The accelerometer was placed at the top of the head, as shown in Figure 2b. Presentation software (Neurobehavioral System Inc., Berkeley, CA, USA) was used to control the presentation and timing of the recorded voice prompts and the beep cue during the experiment, and the signal measurement and execution of the experimental task were synchronized. The 3D coordinates of the light source and detection probe were measured using a 3D magnetic space digitizer (Fastrak, Polhemus Inc., Colchester, VT, USA) to estimate the exact measurement position of the brain area for each individual. The measured 3D coordinates were entered into the ‘register2polhemus’ function, and the ‘depthmap’ function implemented in the NIRS toolbox [28], and the distance from each standard channel to the brain surface was calculated. Based on this distance, the regions of the brain defined by the automated anatomical labeling atlas [29] corresponding to each standard channel were identified.

### 2.5. Cognitive Tests

Five cognitive tests were conducted to identify differences in cognitive function between the three participant groups. As the current study is a cross-sectional comparison, it is impossible to show whether the continued practice of Wellness Darts in older adults affects cognitive function; however, it was preliminarily investigated to confirm its possibility. That is why the statistical tests were conducted between two groups, the expert older and the non-expert older groups. 

#### 2.5.1. Simple Cognitive Test

A simple cognitive (SC) test was used to verify that the participant’s cognitive function had not declined [30]. The SC test is a 50-point cognitive function test that can identify early cognitive decline that cannot be detected by the mini-mental state examination (MMSE), which is often used to examine dementia. The validity and reliability of the SC test has been confirmed for evaluation cognitive ability [30]. A score of fewer than 20 points on the SC test is considered suspicious of cognitive decline. In this study, only participants with a score of 20 or higher were considered to have no cognitive decline and were used in subsequent analyses.

#### 2.5.2. Computational Ability Test

The participants’ computational abilities were measured by administering a test consisting of 30 subtraction problems between two digits, between three and two digits, and between three digits. The differences in mean scores on the computational ability test between the expert older group and the non-expert older group were *t*-tested (*p* < 0.05).

#### 2.5.3. STM 

To evaluate the short-term memory of the participants, a short-term memory (STM) test was conducted on a computer [10]. After the instruction “Please memorize the numbers in order” was displayed on the monitor, nine disordered numbers were displayed on the screen, one by one per second. Then, the participants were instructed to “write the numbers in order,” and they wrote the memorized numbers on a piece of paper during a 20-second time frame. The difference in the mean STM test scores between the expert older group and the non-expert older group was *t*-tested (*p* < 0.05).

#### 2.5.4. CogEvo

CogEvo was used to test the cognitive abilities of the participants; CogEvo is a computer-assisted cognitive function test that assesses disorientation, attention, memory, planning, and spatial awareness [31]. CogEvo can reportedly capture mild changes in cognitive function, and is a simple and convenient ICT tool to assess cognitive changes over time in middle-aged and older adults, as well as during the preclinical stages of dementia [31,32]. CogEvo has 12 different tasks; in this experiment, we selected the flashlight, visual search, number step, and just fit as tasks related to Wellness Darts from the disorientation, attention, memory, planning, and spatial awareness components of CogEvo, respectively. Specifically, the visual search task required participants to click buttons quickly and accurately in a specified sequence (e.g., a–b–c–d…), demanding high levels of attention, sequence processing, and working memory. Therefore, the inclusion of this task indirectly assesses aspects of executive function within the context of cognitive function evaluation in older adults [32]. Also, its validity and reliability has been confirmed for cognitive function evaluation [33]. The difference in the mean CogEvo scores between the expert older group and the non-expert older group was *t*-tested (*p* < 0.05).

#### 2.5.5. TMPT

The trail-making peg test (TMPT) was conducted to assess participants’ cognitive functioning, particularly attentional function and finger dexterity [34]. The TMPT is a peg-movement test that reflects finger dexterity combined with the trail-making test that measures visual attention and executive function [35]. In the TMPT, participants quickly insert 25 cylindrical wooden sticks, called pegs, into numbered holes in numerical order. The board containing the pegs and the board containing the holes were separated. The TMPT has been shown to exhibit sufficient validity and reliability for evaluating cognitive function [36]. The difference in the mean completion times was *t*-tested (*p* < 0.05).

### 2.6. Dart-Throwing Performance Test

To confirm that the dart-throwing skill of the expert older group was higher than that of the control groups, the dart-throwing performances of the three groups were tested. In this test, a target score of 65 points was set and participants were required to throw the darts 10 times and tally their scores to match the target score. The differences in the total dart performance test scores between the expert and younger groups and between the expert and non-expert older groups were *t*-tested (*p* < 0.05). 

### 2.7. Questionnaires

The participants were asked to respond to questions regarding their dominant hand, the number of days per week they practiced Wellness Darts, and the number of years they had been practicing. From these responses, the total number of days of experience for each participant was estimated. 

### 2.8. fNIRS Data Processing

#### 2.8.1. Preprocessing

Previous studies have concluded that HbO in fNIRS is more strongly related to blood oxygenation level-dependent (BOLD) signals measured by functional magnetic resonance imaging (fMRI) than HbR [37,38,39]. fMRI has better spatial resolution than fNIRS, and has been used in many cognitive neuroscience studies. In this study, we used HbO to analyze brain activity. The fNIRS data were preprocessed using the NIRS toolbox [29] to remove physiological noise such as skin blood flow, heart rate, and pulse rate. Optical intensity data acquired from the fNIRS system were converted to optical density (OD) data, and then to Hb concentration data based on the modified Beer–Lambert law [25]. A bandpass filter (0.008–0.09 Hz) was applied to remove noise such as low-frequency drift and heartbeat [39,40].

#### 2.8.2. Activation Analysis

The level of task-induced brain activation for each individual was estimated using the iteratively reweighted least-squares model (AR-IRLS) with short separation (SS) and acceleration, a general linear model (GLM)-based analysis method [27,41]. In the GLM, task-related brain activation was modeled by the hemodynamic response function (HRF). The measured brain activity, i.e., HbO signal, was regressed on the ideal task-related activity calculated from the canonical HRF and a design matrix indicating the type of experimental task, and the partial regression coefficients were estimated via IRLS, a least-squares method. The AR-IRLS allows brain activation analysis to consider the effects of arbitrary confounding factors on brain activity, such as the SS signal measured with a short-distance channel, blood pressure, respiration, and acceleration. In this study, we introduced the SS signal and acceleration data as physiological regressors of the GLM to reduce the effects of skin blood flow and body motion on the HbO signal [27,42].

#### 2.8.3. Hemispheric Lateralization Analysis

The level of hemispheric lateralization was calculated from the lateralization index (LI) shown in Equation (1).

(1) $$LI=\frac{\left|AL-AR\right|}{\left|AL\right|+|AR|}$$

where AL is the sum of β-values in the regions of interest (ROIs) in the left hemisphere, and AR is the sum of those in the right hemisphere [43,44]. We used absolute values for the LI values to allow for the fact that the dominant hemisphere differs depending on the dominant hand. 

### 2.9. Statistical Analysis

#### 2.9.1. Activation Analysis

For individual-level analysis, the following two contrasts were created for the HbO data of the younger group, the expert older group, and the non-expert older group: “rest vs. calculation” and “rest vs. planning”. The β-values for each experimental condition and each channel were calculated. The statistical significance of the task-related brain activity was then verified by testing the null hypothesis that the estimated β coefficient was not significantly different from zero (*t*-test, *p* < 0.05). Note that multiple comparisons were corrected using the Benjamini–Hochberg method [42]. In addition, to test the hypothesis that there is no difference in the level of IPL activation between older adults who continually practiced Wellness Darts and younger adults who were not experienced in Wellness Darts, β-values of the IPL were compared between the three groups (*t*-test, *p* < 0.016, Bonferroni corrected).

Next, for the group-level analysis, we constructed the most consistent contrast images of group-level brain activity using simultaneously the β-values obtained from the individual-level analysis of all participants, based on [28]. To estimate significant group-level activity, we used participants as random effects and conditions (planning, calculation, and rest blocks) as fixed effects. Finally, all of the participants were considered as a sample drawn from the population, and a one-sample *t*-test was used to test if the population mean was greater than zero with respect to the *t*-value. The significance level was set at *p* < 0.05 (false discovery rate corrected at peak level), and the brain regions activated during the calculation and planning blocks were estimated.

#### 2.9.2. Hemispheric Lateralization Analysis

The LI values were used to compare the degree of hemispheric lateralization among the three groups of participants (*t*-test, *p* < 0.016, Bonferroni corrected). In addition, we tested whether there was a positive correlation between the number of days of Wellness Darts experience and LI values in the expert older group (Spearmans’ rank correlation test, *p* < 0.05).

## 3. Results

### 3.1. Descriptive Statistics on Three Groups

The descriptive statistics on the three groups, including age, days of practicing Wellness Darts, cognitive test scores, and scores of dart-throwing performance tests, are summarized in Table 1. The following subsections describe the details of the results, including statistical tests.

### 3.2. fNIRS Data

#### 3.2.1. Hemispheric Lateralization Analysis

**Figure 3:**
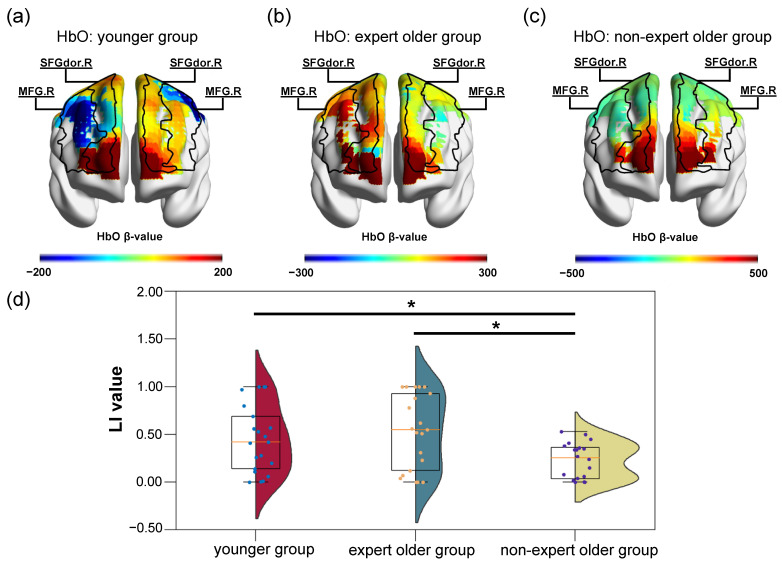
β maps and LI values in the planning block. (**a**–**c**) β maps of participants with LI values close to the median in younger, expert older, and non-expert older groups are shown, respectively. (**d**) Distribution of LI values for the three participant groups. * *p* < 0.016. β maps were visualized using the xjView toolbox and BrainNet viewer.

**Figure 4:**
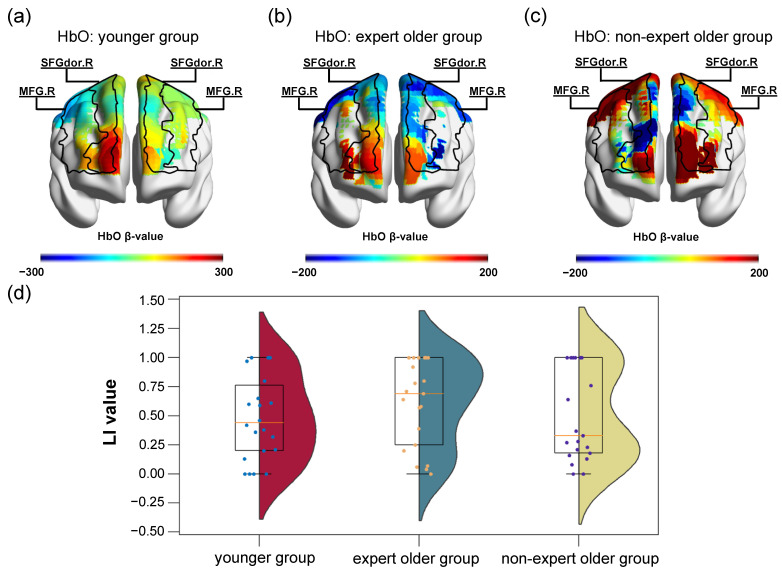
β maps and LI values in the calculation block. (**a**–**c**) β maps of participants with LI values close to the median in the younger, expert older, and non-expert older groups are shown, respectively. (**d**) Distribution of LI values for the three participant groups. β maps were visualized using the xjView toolbox and BrainNet viewer.

We hypothesized that the LI values of the younger group and the expert older group would be larger than those of the non-expert older group. LI values were calculated from the respective β-values of the two task blocks (planning and calculation) for the three participant groups. The LI values in the planning and calculation blocks of the younger group were 0.45 ± 0.34 and 0.49 ± 0.35, respectively. The LI values in the planning and calculation blocks of the expert older group were 0.53 ± 0.38 and 0.61 ± 0.35, respectively. Finally, for the non-expert older group, they were 0.23 ± 0.18 and 0.50 ± 0.38 in the planning and calculation blocks, respectively. The box plots of the LI values in the planning and calculation blocks for the three groups of participants are shown in Figure 3d and Figure 4d, respectively. Furthermore, the β maps of the participants who showed the closest values to the median in the LI values for each group of participants are shown in Figure 3a–c and Figure 4a–c.

There were no significant differences between the LI values of the younger group and the expert older group in the planning block (*t* = 0.6730, *p* = 0.504, *d* = 0.213). On the other hand, in the planning block, the LI values of the expert older group were significantly greater than those of the non-expert older group (*t* = 3.188, *p* = 0.003, *d* = 1.005). Furthermore, the LI values of the younger group were significantly greater than those of the non-expert older group in the planning block (*t* = 2.58, *p* = 0.014, *d* = 0.815).

In the calculation block, there were no differences in LI values between the younger group and the expert older group (*t* = 1.009, *p* = 0.319, *d* = 0.319). Similarly, there were no differences in the LI between the expert older group and the non-expert older group (*t* = 0.828, *p* = 0.413, *d* = 0.262). There were also no differences in LI values between the younger group and the non-expert older group (*t* = 0.144, *p* = 0.886, *d* = 0.046).

**Figure 5:**
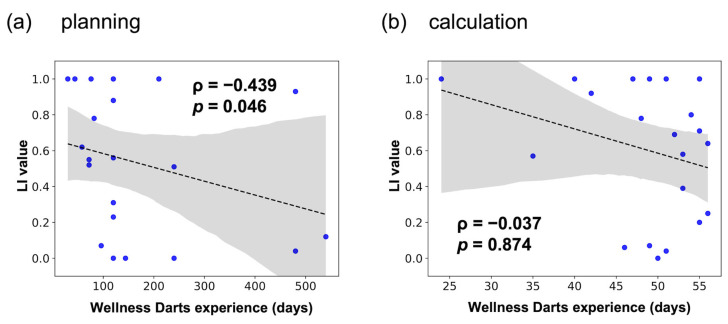
Correlation between the number of days of Wellness Darts experience and LI values in the expert older group is indicated as a scatter plot (blue points), with a fitted regression line (dashed line) and 95% confidence interval band (gray shadow). (**a**) Planning block. (**b**) Calculation block.

We also hypothesized that the number of days of Wellness Darts experience in the expert older group would be positively correlated with LI values. The results of the Spearman’s rank correlation test between the number of days of Wellness Darts experience and LI values showed a negative significant correlation for the planning block (*ρ* = −0.439, *p* = 0.046, Figure 5a) but no significant correlation for the calculation block (*ρ* = −0.037, *p* = 0.874, Figure 5b).

**Figure 6:**
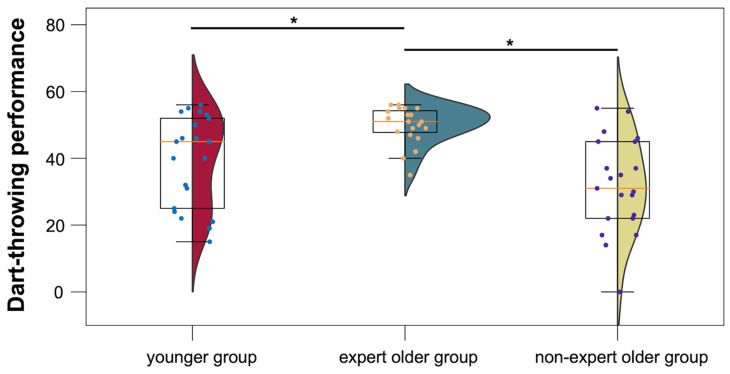
Dart performance levels in the younger, expert older, and non-expert older groups. * *p* < 0.05.

To investigate the Wellness Darts experience of the expert older group in terms of skill, the mean darts performance test score (target score was 65 points; the closer the total score of 10 dart throws was to 65 points, the better the skill) was compared between the three participant groups. The mean scores for the younger group, the expert older group, and the non-expert older group were 39.3 ± 13.4, 49.9 ± 5.5, and 31.9 ± 13.7, respectively. There were no participants who exceeded the 65 points. As shown in Figure 6, the expert older group showed better dart performance compared to the younger group (*t* = 3.247, *p* = 0.003, *d* = 1.022). Similarly, the expert older group showed better performance than the non-expert older group (*t* = 5.400, *p* = 0.00001, *d* = 1.700).

**Figure 7:**
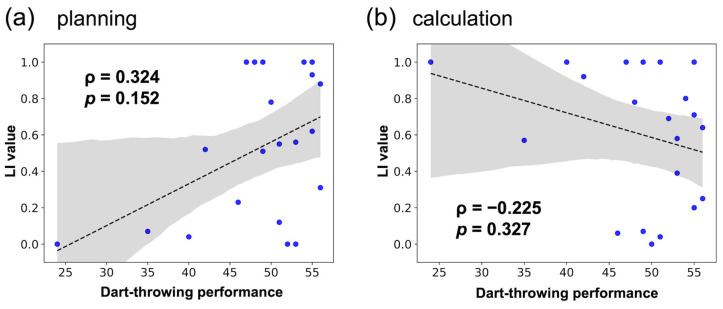
Correlation between darts performance level and LI value in the expert older group. (**a**) Planning block. (**b**) Calculation block.

Furthermore, inspired by the results of no significant ‘positive’ correlation between the number of days of Wellness Darts experience and LI values, we assumed that dart proficiency could be assessed not only by the number of days of experience, but also by dart-throwing performance. Therefore, a positive correlation between the total dart performance test score and the degree of hemispheric lateralization of brain activity in the expert older group was also tested (Spearman’s rank correlation test, *p* < 0.05). It should be noted that this is an exploratory analysis based on the results shown in Figure 5. However, no significant correlation between the total dart performance test score and the LI value was confirmed (planning block: *ρ* = 0.324, *p* = 0.152, Figure 7a; calculation block: *ρ* = −0.225, *p* = 0.327, Figure 7b).

#### 3.2.2. Activation Analysis

**Figure 8:**
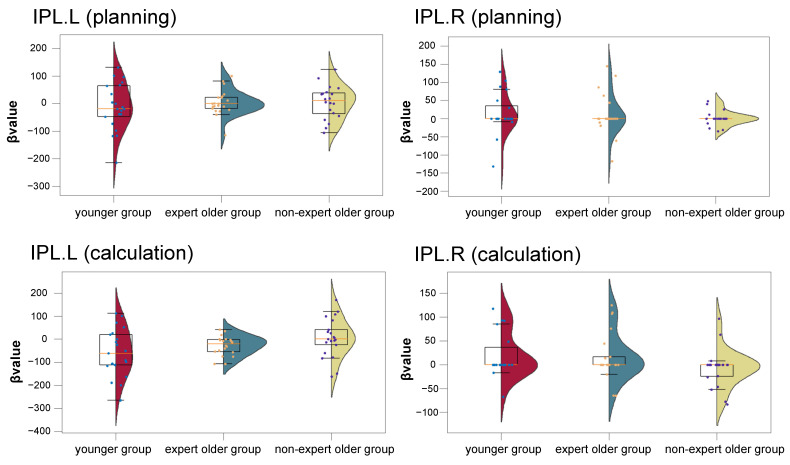
β-values of the IPL in the younger, expert older, and non-expert older groups.

The activation analysis shown in Figure 8 indicates that in the planning block, there was no difference between the left and right IPL (IPL.L: *t* = 0.738, *p* = 0.466, *d* = 0.234. IPL.R: *t* = 0.134, *p* = 0.894, *d* = 0.043). Similarly, there was no difference between the expert older group and the non-expert older group (IPL.L: *t* = 0.042, *p* = 0.966, *d* = 0.014; IPL.R: *t* = 0.821, *p* = 0.419, *d* = 0.257). Furthermore, there was no difference between the younger group and the non-expert older group (IPL.L: *t* = 0.711, *p* = 0.481, *d* = 0.226. IPL.R: *t* = 0.967, *p* = 0.343, *d* = 0.312).

In the calculation block, there was no difference between the left and right IPL (IPL.L and IPL.R) of the younger group and the expert older group (IPL.L: *t* = 1.211, *p* = 0.236, *d* = 0.381; IPL.R: *t* = 0.206, *p* = 0.838, *d* = 0.068). Similarly, there was no difference between the expert older group and the non-expert older group (IPL.L: *t* = 1.625, *p* = 0.115, *d* = 0.513; IPL.R: *t* = 1.642, *p* = 0.109, *d* = 0.523). There was no difference between the younger group and the non-expert older group (IPL.L: *t* = 2.176, *p* = 0.036, *d* = 0.688. IPL.R: *t* = 1.844, *p* = 0.074, *d* = 0.621).

Supplementary Tables S1–S6 summarize the results of individual-level analysis and show the regions of interest where the *p*-values are less than 0.05. Table 2, Table 3 and Table 4 summarize the results of group-level analysis and show only the ROIs. There was significant activation in the ROIs in the individual-level analysis in the expert older group, but no significant activation in the group-level analysis.

### 3.3. Cognitive Tests

#### 3.3.1. Computational Ability Test

**Figure 9:**
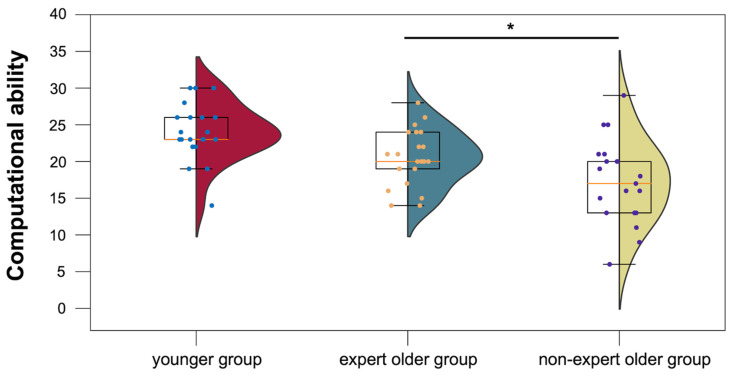
Computational ability test scores for the younger, expert older, and non-expert older groups. * *p* < 0.05.

The mean computational test scores for the younger group, the expert older group, and the non-expert older group were 24.0 ± 3.8, 20.5 ± 3.8, and 17.1 ± 5.5, respectively. As shown in Figure 9, the expert older group had higher scores on the computational skills test than the non-expert older group (*t* = 2.275, *p* = 0.029, *d* = 0.719).

#### 3.3.2. STM Test

**Figure 10:**
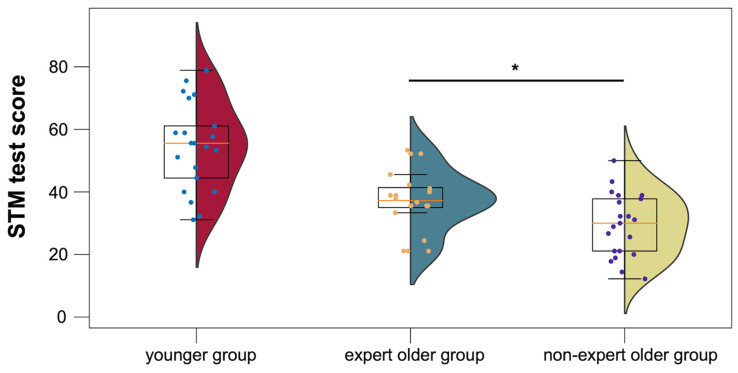
STM test scores for the younger, expert older, and non-expert older groups. * *p* < 0.05.

The mean STM test scores of the younger group, the expert older group, and the non-expert older group were 74.6 ± 13.6, 57.1 ± 9.5, and 49.4 ± 9.9, respectively. As shown in Figure 10, the expert older group showed higher STM test scores than the non-expert older group (*t* = 2.475, *p* = 0.018, *d* = 0.792).

#### 3.3.3. CogEvo

**Figure 11:**
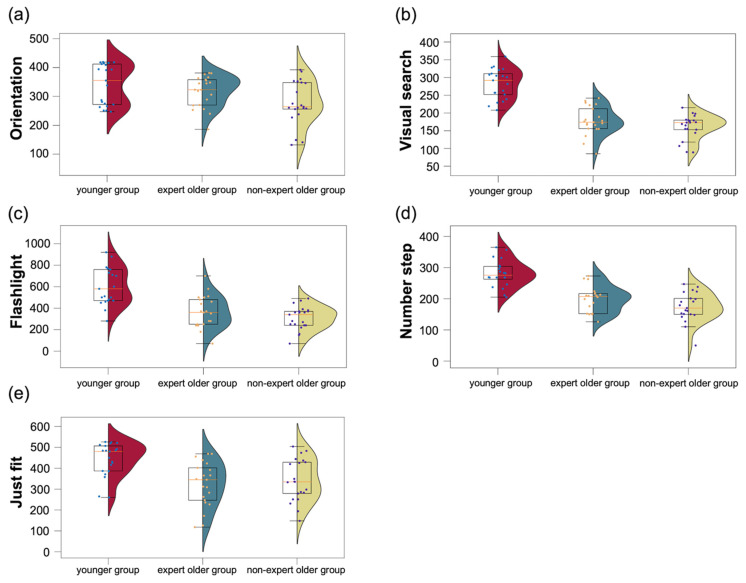
Scores on five different CogEvo tasks for the three groups of participants. (**a**) Orientation. (**b**) Visual search. (**c**) Flashlight. (**d**) Number step. (**e**) Just Fit.

The scores of the five CogEvo tasks for each group of participants are summarized in Figure 11. In the expert older and non-expert older groups, there were no significant differences in the CogEvo scores (disorientation: *t* = 1.892, *p* = 0.067, *d* = 0.598, visual search: *t* = 1.435, *p* = 0.159, *d* = 0.454; flashlight: *t* = 1.386, *p* = 0.174, *d* = 0.438; number step: *t* = 1.722, *p* = 0.093, *d* = 0.550; just fit: *t* = 0.625, *p* = 0.534, *d* = 0.198). 

#### 3.3.4. TMPT

**Figure 12:**
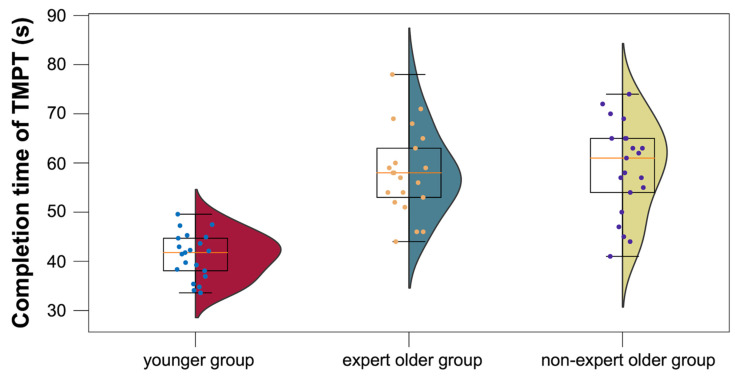
TMPT completion times for the younger, expert older, and non-expert older groups.

The mean TMPT completion times for the younger, expert older, and non-expert older groups were 41.1 ± 4.5, 58.1 ± 8.5, and 58.9 ± 9.2, respectively, and they are summarized in Figure 12. There was no significant difference in TMPT completion time between the older and non-expert older groups (*t* = 0.272, *p* = 0.787, *d* = 0.086).

## 4. Discussion

### 4.1. Hypothesis 1

As seen in the results of the individual-level analysis in Supplementary Tables S1–S6, we were able to confirm the activation of the SFGdor and MFG in the three participant groups. However, there was no significant activation of these regions in the group-level analysis, as shown in Table 2, Table 3 and Table 4. This may be because the mean activation level was estimated to be lower after averaging the results of the group as the individual activated areas varied even in the same region due to the distinct differences in the arrangement of measurement channels in the fNIRS device. 

On the other hand, Figure 3d shows that the LI values of the younger group and the expert older group were significantly higher than those of the non-expert older group in the planning block. In addition, there was no difference in LI values between the younger group and the expert older group. This indicates that the brain activation patterns of participants in the younger group and the expert older group are hemispherically lateralized during the planning of dart throwing and while determining where to hit. The β-maps in Figure 3a–c also show that the activation levels in the younger and expert older groups were higher in one hemisphere, while the non-expert older group showed no hemispheric difference. In the non-expert older group, there is no difference between the hemispheres, and the bilateral activation levels of the SFGdor and MFG are high. This result is consistent with a previous study in which left–right hemispherical asymmetry increased when cognitive training was performed in healthy older adults [15]. In summary, although the SFGdor and MFG were not significantly activated in the expert older group in group-level analysis, Hypothesis 1 was established during the planning of dart throwing, suggesting that continual Wellness Darts training promotes hemispheric lateralization in brain activity in older adults. These results suggest that the Wellness Darts game holds potential for improving cognitive function.

### 4.2. Hypothesis 2

Figure 8 indicates that there were no significant differences in the β-values of the left and right IPL between the younger group and the expert older group. Further, there was no significant difference in the β-values of the left and right IPL between the younger and non-expert older groups, and between the expert and non-expert older groups. However, in the three participant groups in the present study, the IPL activation was comparable, suggesting no difference in IPL activation between the older groups. The previous study to which the hypothesis refers involved healthy subjects and patients with MCI and Alzheimer’s disease [22]. The participants in the present study were only healthy adults, suggesting that the difference in cognitive function between the older groups and the younger group did not affect IPL activation.

Given fNIRS’s limitations in spatial resolution, pinpointing the exact reasons for the differences in IPL activation among our participant groups is challenging. While fNIRS is advantageous for non-invasiveness and suitability for experimenting with physical activity, it may need spatial resolution to capture differences in IPL activation. Considering this, we can effectively optimize the placement of fNIRS channels to improve the higher spatial resolution around the IPL; densely clustering fNIRS channels around the IPL may provide more precise data. Alternatively, functional magnetic resonance imaging (fMRI) could be used, which has a higher spatial resolution than fNIRS. fMRI could provide more detailed brain activity mapping, potentially revealing differences among the groups that were not detectable with fNIRS. However, it should be noted that since fMRI imposes limitations on a participant’s movements, the current experimental design should be improved to simulate dart throwing. Nonetheless, a follow-up study using fMRI to investigate IPL activation across three healthy groups would be a valuable step forward.

### 4.3. Hypothesis 3

As shown in Figure 5, there was no positive correlation between the number of days of Wellness Darts experience and the LI value of the expert older group; therefore, Hypothesis 3 was not supported. On the other hand, in Figure 6, the dart performance of the expert older group was higher than that of the other two groups. In addition, Figure 7 implies a positive correlation between dart performance and LI values in the planning block, although it is not statistically significant. On the other hand, the total score of our dart performance test indicates the accuracy of dart throwing, which is improved by the number of days of experience. Therefore, the results of Figure 6 and Figure 7 may contribute to the validity of Hypothesis 3. It also suggests that factors beyond the scope of our current study, such as individual differences in cognitive reserve or the variability in engagement levels with the Wellness Darts program, could impact the strength and direction of the observed correlations. Incorporating a more detailed assessment of participants’ engagement with the Wellness Darts program, including qualitative aspects of their experience, may provide valuable insights into how different components of the program contribute to cognitive health. Also, since the current results suggested weak correlations, the correlations should be tested again with a larger sample size. These issues would be investigated in future longitudinal controlled studies of the Wellness Darts intervention that measure dart performance levels to verify the increase in LI values promoted by Wellness Darts training.

### 4.4. Cognitive Tests

In addition to hypotheses testing, the results of the three cognitive function tests to determine the impact of Wellness Darts on cognitive function are also discussed.

#### 4.4.1. Computational Ability Test

The score of the expert older group was significantly higher than that of the non-expert older group. In other words, the expert older group had better computational ability than the non-expert older group. According to previous studies, cognitive function, particularly for high-load tasks, declines as the brain deteriorates with age [45]. Therefore, the results of the computational ability test in this study suggest that continual practice of Wellness Darts possibly reduces the decline in calculation ability due to aging.

#### 4.4.2. STM Test

The STM test scores of the expert older group were higher than those of the non-expert older group, and the difference was significant. Fandakova et al. [46] have shown that short-term memory capacity declines with age. Therefore, our results indicate that constant training with Wellness Darts possibly reduces the age-related decline in short-term memory capacity.

#### 4.4.3. CogEvo

No significant differences were found in the scores between the expert older group and the non-expert older group on the five CogEvo tasks. Although CogEvo has been reported to be able to significantly distinguish between Alzheimer’s disease, MCI, and cognitively normal groups of older adults [31,32], our results showed that the expert older group and the non-expert older group did not differ significantly in their scores on the five CogEvo tasks. In fact, our results indicated that the expert older and non-expert older groups had similar cognitive levels. In other words, the older groups in our experiment had normal levels of cognitive function, or the differences in cognitive function were at levels undetectable by CogEvo. Another explanation could be that the older groups were unfamiliar with the operation of CogEvo, as it was conducted using a tablet device.

#### 4.4.4. TMPT

As seen in Figure 12, there was no significant difference in the completion time of the TMPT between the expert older and the non-expert older groups. The TMPT requires skill from the participant as a component of physical function. We expected that participants engaged in ongoing Wellness Darts exercises would demonstrate improved skillfulness; however, in this cross-sectional study, no differences were observed between the expert older and non-expert older groups. A previous longitudinal study that divided older adults into a dual-task (DT) exercise group and a control group observed a significant difference in the TMPT before and after intervention in the DT exercise group, but no significant TMPT difference was identified between the two groups [47]. Due to the pilot nature of the current study based on a cross-sectional comparison, a possible future direction could be to investigate whether the Wellness Darts intervention contributes to improving the TMPT completion time in longitudinal randomized controlled trials.

#### 4.4.5. Strengths of the Current Study, Limitations, and Future Directions

Based on our findings, only Hypothesis 1 was supported in the planning of dart throwing and in determining where to hit. Due to the pilot nature of this study, the sample size was limited, and the effects of several unseen parameters such as education level, socio-economic factors, body mass index, and smoking were not controlled and investigated. These limitations make the current study insufficient to demonstrate that Wellness Darts truly affected brain lateralization. An ANCOVA or GLM analysis that considers control variables would be helpful in this situation. However, despite these limitations, the current study revealed that older adults with Wellness Darts experience have a higher tendency toward hemispheric lateralization than those with no experience. The current results are valuable in motivating us to proceed to future longitudinal randomized controlled trials (RCTs) to explore the effects of continual Wellness Darts training on hemispheric lateralization with a larger sample size.

Additional limitations need to be acknowledged in the current study. Due to constraints in resources and time, we did not conduct preliminary screenings for global cognitive functioning in the non-expert older group. This means that the health status of participants within this group remains undefined, which could potentially influence the interpretation of our findings. We advocate for the inclusion of comprehensive cognitive function screenings for all participants in future research.

When reflecting on our methodological choices, we should recognize that the Montreal Cognitive Assessment (MoCA) could have offered a more comprehensive evaluation than the SC test, particularly in executive functions. We realize the potential benefits of incorporating the MoCA for future longitudinal studies to obtain a more detailed assessment of cognitive functions.

Moreover, it is essential to acknowledge that strict blinding was not feasible due to the nature of the Wellness Darts intervention. The roles of analysts and experimenters overlapped significantly, and participants’ expertise levels in darts were visually identifiable. Furthermore, the age-related differences among groups were apparent, complicating any attempt at blinding. These factors potentially introduce biases that are difficult to quantify. Despite these challenges, efforts were made to mitigate bias through critical internal review and anonymization of data before analysis. Recognizing these constraints, future studies should explore methodologies to enable more rigorous blinding and minimize biases. Future longitudinal RCTs, including the blinding process, will also allow us to reduce these biases.

Lastly, the method used for participant recruitment may have introduced potential selection bias and impacted the validity of our findings. Specifically, participants in the older expert group were recruited from a specific community sports club, employing a convenience sampling approach. In contrast, the non-expert older participants were recruited using non-probabilistic methods. This might result in differences in the baseline characteristics between the groups beyond their experience with Wellness Darts. This issue should be addressed in future experiments.

## 5. Conclusions

Wellness Darts is a cognitively challenging sport that combines exercise and calculation. In this research, we aimed to cross-sectionally confirm the difference in hemispheric lateralization between expert and non-expert players as a pilot study prior to the longitudinal randomized controlled trials. The results showed that the younger and the expert older groups had significantly higher LI values than the non-expert older group, and that these values did not differ between the expert older and the younger groups. These results suggest that playing the Wellness Darts game possibly promotes hemispheric lateralization.

## Figures and Tables

**Table 1:** Descriptive statistics on the three groups.

Characteristics		Expert Older Group	Non-Expert Older Group (Older Control)	Younger Group (Non-Expert Younger Control)
Age (years)		75.1 ± 4.3	74.1 ± 4.5	22.5 ± 0.8
Number of days of Wellness Darts experience (days)		170.7 ± 145.6	0.0	0.0
Cognitive tests	SC test	41.1 ± 7.8	44.5 ± 5.8	48.8 ± 2.5
	Computational ability test	20.5 ± 3.8	17.1 ± 5.5	24.0 ± 3.8
	STM test	57.1 ± 9.5	49.4 ± 9.9	74.6 ± 13.6
	TMPT	58.1 ± 8.5	58.9 ± 9.2	41.1 ± 4.5
	CogEvo: disorientation	361.8 ± 52.7	278.2 ± 74.5	341.5 ± 68.8
	CogEvo: visual search	177.9 ± 39.0	161.3 ± 33.8	282.0 ± 41.3
	CogEvo: flashlight	364.3 ± 146.9	308.1 ± 106.4	604.3 ± 169.2
	CogEvo: number step	197.4 ± 40.8	173.5 ± 45.9	280.9 ± 42.8
	CogEvo: just fit	332.2 ± 104.9	342.4 ± 98.7	442.1 ± 78.2
Dart-throwing performance		49.9 ± 5.5	31.9 ± 13.7	39.3 ± 13.4

**Table 2:** Task-related activation in the region of interest revealed by group-level analysis (younger group).

Condition	Anatomical Region	*t*-Value	*p*-Value
			(FDR-Corrected)
planning	IPL.L	0.15	0.909
	MFG.L	1.931	0.186
	MFG.R	3.321	0.022
	SFGdor.L	3.155	0.022
	SFGdor.R	3.822	0.012
calculation	IPL.L	0.139	0.454
	MFG.L	3.321	0.022
	MFG.R	3.321	0.022
	SFGdor.L	2.866	0.052
	SFGdor.R	5.847	5.72 × 10^−5^

Abbreviations: IPL: Parietal Inf lobule; MFG: middle frontal gyrus; SFGdor: dorsal superior frontal gyrus; R: right; L: left.

**Table 3:** Task-related activation in the region of interest revealed by group-level analysis (expert older group).

Condition	Anatomical Region	*t*-Value	*p*-Value
			(FDR-Corrected)
planning	IPL.L	0.127	0.988
	IPL.R	1.706	0.548
	MFG.L	−0.163	0.988
	MFG.R	1.238	0.709
	SFGdor.L	0.343	0.988
	SFGdor.R	1.040	0.817
calculation	IPL.L	0.545	0.723
	IPL.R	0.367	0.820
	MFG.L	0.999	0.495
	MFG.R	1.542	0.289
	SFGdor.L	1.115	0.272
	SFGdor.R	0.989	0.495

Abbreviations: IPL: Parietal Inf lobule; MFG: middle frontal gyrus; SFGdor: dorsal superior frontal gyrus; R: right; L: left.

**Table 4:** Task-related activation in the region of interest revealed by group-level analysis (non-expert older group).

Condition	Anatomical Region	*t*-Value	*p*-Value
			(FDR-Corrected)
planning	IPL.L	2.282	0.385
	MFG.L	0.446	0.943
	MFG.R	−0.928	0.794
	SFGdor.L	−0.331	0.943
	SFGdor.R	−0.684	0.904
calculation	IPL.L	1.234	0.525
	MFG.L	0.309	0.876
	MFG.R	−1.118	0.540
	SFGdor.L	−0.496	0.803
	SFGdor.R	−0.469	0.815

Abbreviations: IPL: Parietal Inf lobule; MFG: middle frontal gyrus; SFGdor: dorsal superior frontal gyrus; R: right; L: left.

## Data Availability

The datasets generated during and/or analyzed during the current study are available from the corresponding author upon reasonable request.

## Supplementary Materials

The following supporting information can be downloaded at: https://www.mdpi.com/article/10.3390/healthcare12070734/s1, Table S1. Brain regions that showed task-related activation for each individual in the planning block (younger group). Only regions of interest that showed *p* < 0.05 (FDR corrected at peak level) are shown.; Table S2. Brain regions that showed task-related activation for each individual in the calculation block (younger group). Only regions of interest that showed *p* < 0.05 (FDR corrected at peak level) are shown.; Table S3. Brain regions that showed task-related activation in the planning block for each individual (expert older group). Only regions of interest with *p* < 0.05 (FDR corrected at peak level) are shown.; Table S4. Brain regions that showed task-related activation for each individual in the calculation block (expert older group). Only regions of interest with *p* < 0.05 (FDR corrected at peak level) are shown.; Table S5. Brain regions that showed task-related activation for each individual in the planning block (non-expert older group). Only regions of interest with *p* < 0.05 are shown.; Table S6. Brain regions that showed task-related activation for each individual in the calculation block (non-expert older group). Only regions of interest with *p* < 0.05 are shown.
