# Supplementary material for: Hemispheric Lateralization in Older Adults Who Habitually Play Darts: A Cross-Sectional Study Using Functional Near-Infrared Spectroscopy
Source: Healthcare (Basel). 2024 Mar 27;12(7):734. doi: 10.3390/healthcare12070734 (PMC11012225; doi:10.3390/healthcare12070734)
Supplement: Supplementary file 1 [file healthcare-12-00734-s001.zip › healthcare-2893688-supplementary.pdf]

Supplementary Materials

Table S1. Brain regions that showed task-related activation for each individual in the planning block (younger group). Only regions of interest that showed p < 0.05 (FDR corrected at peak level) are shown.

Participant	Anatomical Region	t-value	p-value (FDR-corrected)	Participant	Anatomical Region	t-value	p-value (FDR-corrected)
1	IPL.L	3.765	5.76E-04	11	MFG.L	5.449	2.54E-07
	MFG.L	4.408	4.06E-05		MFG.R	8.413	8.76E-16
	MFG.R	10.33	5.57E-23		SFGdor.L	6.024	1.12E-08
	SFGdor.L	7.434	1.48E-12		SFGdor.R	8.779	5.39E-17
	SFGdor.R	9.248	9.70E-19	12	MFG.L	4.957	1.77E-06
2	IPL.R	-4.224	3.05E-04		MFG.R	5.836	2.56E-08
	MFG.L	2.498	0.038		SFGdor.L	6.686	1.67E-10
	MFG.R	-2.941	0.012		SFGdor.R	5.824	2.63E-08
	SFGdor.R	-2.978	0.012	13	IPL.L	-2.443	0.035
3	IPL.R	3.342	0.002		MFG.L	5.219	1.93E-06
	MFG.R	6.801	1.32E-10		SFGdor.L	5.193	2.03E-06
	SFGdor.R	5.896	2.37E-08	14	MFG.R	7.688	3.71E-13

4	IPL.L	-3.846	1.84E-04		SFGdor.R	6.225	2.75E-09
	IPL.R	-4.344	2.73E-05	15	IPL.L	-3.952	2.21E-04
	MFG.L	-6.759	7.67E-11		MFG.R	8.127	8.13E-15
	MFG.R	-2.298	0.028		SFGdor.R	9.337	7.87E-19
	SFGdor,L	-4.55	1.07E-05	16	IPL.L	-6.443	3.36E-09
	SFGdor.R	-2.273	0.029		MFG.R	2.473	0.026
5	MFG.L	8.83	9.44E-18	17	IPL.L	-4.459	9.81E-05
	MFG.R	6.576	2.80E-10		SFGdor.L	-2.842	0.02
	SFGdor.L	19.18	2.87E-77	18	IPL.L	-9.742	1.12E-20
	SFGdor.R	7.335	1.45E-12		MFG.L	-3.147	0.005
6	IPL.R	6.645	3.50E-10		MFG.R	-3.923	3.48E-04
	SFGdor.L	-4.996	2.78E-06		SFGdor.L	-5.72	7.17E-08
	SFGdor.R	2.399	0.028		SFGdor.R	-4.389	5.23E-05
7	IPL.L	2.771	0.008	19	IPL.L	6.362	7.05E-10
	IPL.R	4.191	6.82E-05		MFG.L	7.276	2.11E-12
	MFG.R	4.289	4.64E-05		MFG.R	7.561	3.03E-13
	SFGdor.L	4.583	1.38E-05		SFGdor.L	6.035	4.98E-09

	SFGdor.R	4.365	3.38E-05		SFGdor.R	10.65	3.69E-24
8	IPL.L	4.411	1.56E-05	20	MFG.R	4.538	1.88E-05
	IPL.R	4.257	3.09E-05		SFGdor.R	4.636	1.26E-05
	MFG.L	5.811	1.70E-08	21	IPL.L	2.346	0.033
	MFG.R	5.317	2.31E-07		MFG.L	3.582	9.94E-04
	SFGdor.L	9.745	5.83E-21		MFG.R	12.54	9.28E-34
	SFGdor.R	6.694	7.43E-11		SFGdor.L	4.791	5.92E-06
10	IPL.L	3.432	0.002		SFGdor.R	12.01	3.62E-31
	MFG.L	3.113	0.004				
	MFG.R	4.145	1.42E-04				
	SFGdor.R	3.909	3.05E-04				

Abbreviations: IPL: Parietal Inf lobule; MFG: middle frontal gyrus; SFGdor: dorsal superior frontal gyrus; R: right; L: Left.

Table S2. Brain regions that showed task-related activation for each individual in the calculation block (younger group). Only regions of interest that showed p < 0.05 (FDR corrected at peak level) are shown.

Participant	Anatomical Region	t-value	p-value (FDR-corrected)	Participant	Anatomical Region	t-value	p-value (FDR-corrected)
1	MFG.R	7.509	2.55E-12	11	IPL.L	-3.342	0.002

	SFGdor.L	4.194	2.04E-04		MFG.L	5.89	1.75E-08
	SFGdor.R	5.533	7.67E-05		MFG.R	6.951	2.72E-11
2	IPL.R	-8.217	1.51E-14		SFGdor.L	6.464	6.57E-10
2	MFG.R	-3.969	8.20E-04		SFGdor.R	8.092	8.43E-15
2	SFGdor.R	-5.505	7.29E-07	13	IPL.L	-2.444	0.035
3	IPL.L	-5.429	8.59E-07	13	MFG.L	5.219	1.93E-06
3	MFG.R	5.85	8.88E-08	13	SFGdor.L	5.193	2.03E-06
3	SFGdor.R	2.523	0.023	14	MFG.L	3.061	0.004
4	IPL.L	-8.516	7.35E-17	14	MFG.R	7.079	9.36E-12
4	IPL.R	-9.993	1.64E-22	14	SFGdor.L	3.61	6.08E-04
4	MFG.L	-2.591	0.011	14	SFGdor.R	6.778	6.56E-11
4	MFG.R	-2.234	0.029	15	MFG.R	7.157	1.92E-11
4	SFGdor.L	-5.707	2.23E-08	15	SFGdor.R	8.688	2.59E-16
4	SFGdor.R	-4.508	9.39E-06	16	IPL.L	-6.433	2.05E-09
5	IPL.L	-3.004	0.007	17	IPL.L	-6.99	4.20E-11
5	MFG.L	6.701	1.05E-10	17	MFG.L	-7.918	5.44E-14
5	MFG.R	2.79	0.013	17	SFGdor.L	-8.635	2.21E-16

	SFGdor.L	15.949	2.31E-54		SFGdor.R	-3.074	0.006
	SFGdor.R	2.956	0.008		IPL.L	-4.03	1.73E-04
6	IPL.R	3.981	2.01E-04	18	MFG.L	-5.082	2.31E-06
	MFG.R	3.87	2.90E-04		MFG.R	-3.547	9.51E-04
	SFGdor.L	-5.067	2.42E-06		SFGdor.L	-6.342	2.48E-09
	SFGdor.R	4.977	3.63E-06		SFGdor.R	-4.291	6.13E-05
7	IPL.L	3.011	0.009	19	IPL.L	3.566	6.62E-04
	IPL.R	4.247	1.77E-04		IPL.R	-2.674	0.011
8	IPL.L	3.933	1.30E-04		MFG.L	4.345	3.13E-05
	IPL.R	3.329	0.001		MFG.R	7.278	1.86E-12
	MFG.L	5.935	7.59E-09		SFGdor.L	4.855	3.25E-06
	MFG.R	2.072	0.044		SFGdor.R	11.48	2.56E-28
	SFGdor.L	8.248	2.22E-15	20	IPL.L	-5.517	6.64E-08
	SFGdor.R	2.922	0.004		MFG.L	-2.113	0.042
9	IPL.L	-3.998	1.99E-04		MFG.R	4.332	2.16E-05
	IPL.R	-5.626	1.14E-07		SFGdor.R	3.732	2.57E-04
	MFG.L	-3.412	0.001	21	MFG.L	3.828	3.12E-04

	MFG.R	14.12	6.09E-42
	SFGdor.L	3.692	4.94E-04
	SFGdor.R	12.36	3.38E-33

Abbreviations: IPL: Parietal Inf lobule; MFG: middle frontal gyrus; SFGdor: dorsal superior frontal gyrus; R: right; L: Left.

Table S3. Brain regions that showed task-related activation in the planning block for each individual (expert older group). Only regions of interest with $p < 0.05$ (FDR corrected at peak level) are shown.

Participant	Anatomical Region	t-value	p-value (FDR-corrected)	participant	Anatomical Region	t-value	p-value (FDR-corrected)
1	IPL.R	4.418	4.97E-05	12	MFG.L	-5.516	1.33E-06
	MFG.R	-4.478	3.88E-05		MFG.R	-4.304	3.30E-04
2	IPL.L	5.142	1.98E-06	13	MFG.L	6.548	1.97E-10
	IPL.R	4.167	1.24E-04		MFG.R	3.212	0.002
	MFG.L	-4.8	9.13E-06	14	MFG.L	5.044	3.19E-06
	SFGdor.L	-3.54	0.001		MFG.R	6.031	2.05E-08
3	MFG.L	-7.369	1.65E-12		SFGdor.L	4.537	2.84E-05
	SFGdor.L	-4.309	4.51E-05		SFGdor.R	2.859	0.009
4	IPL.R	-5.123	1.03E-06	15	IPL.L	6.276	3.28E-09

	MFG.L	4.82	4.32E-06		IPL.R	7.845	1.55E-13
5	IPL.R	2.806	0.01		MFG.L	4.419	3.04E-05
	SFGdor.L	-3.32	0.002		MFG.R	5.777	6.03E-08
6	MFG.L	-2.802	0.014		SFGdor.R	4.734	8.11E-06
7	MFG.L	4.296	1.29E-04	16	IPL.R	-4.795	2.22E-05
	SFGdor.L	3.352	0.003	17	IPL.L	-4.806	5.20E-06
8	IPL.L	-4.035	2.56E-04		MFG.L	-2.944	0.006
	SFGdor.R	-2.635	0.02	19	MFG.L	-2.989	0.007
9	IPL.L	3.995	4.11E-04		MFG.R	-5.129	1.74E-06
	SFGdor.L	3.754	9.62E-04		SFGdor.L	-4.712	1.02E-05
10	IPL.L	-3.172	0.007		SFGdor.R	-2.744	0.012
	IPL.R	7.868	2.43E-13	20	MFG.L	2.756	0.023
	MFG.R	2.39	0.049		SFGdor.L	3.671	0.001
11	MFG.R	7.11	3.42E-11	21	MFG.R	3.168	0.005
	SFGdor.R	4.948	1.11E-05		SFGdor.R	5.267	1.52E-05

Abbreviations: IPL: Parietal Inf lobule; MFG: middle frontal gyrus; SFGdor: dorsal superior frontal gyrus; R: right; L: Left.

Table S4. Brain regions that showed task-related activation for each individual in the calculation block (expert older group). Only regions of interest with $p < 0.05$ (FDR corrected at peak level) are shown.

Participant	Anatomical Region	t-value	p-value (FDR-corrected)	Participant	Anatomical Region	t-value	p-value (FDR-corrected)
1	IPL.R	4.418	4.97E-05	13	MFG.L	5.359	2.13E-07
	MFG.R	-4.478	3.88E-05		MFG.R	5.988	6.77E-09
2	IPL.L	5.142	1.98E-06		SFGdor.L	3.457	9.19E-04
	IPL.R	4.167	1.24E-04		SFGdor.R	3.344	0.001
	MFG.L	-4.8	9.13E-06	14	MFG.R	3.831	4.78E-04
	SFGdor.L	-3.54	0.001	15	IPL.L	8.888	1.50E-17
3	MFG.L	-7.369	1.65E-12		IPL.R	7.401	1.02E-12
	SFGdor.L	-4.309	4.51E-05		MFG.L	6.731	8.57E-11
4	IPL.R	-5.123	1.03E-06		MFG.R	8.754	3.76E-17
	MFG.L	4.82	4.32E-06		SFGdor.L	5.877	1.52E-08
5	IPL.R	2.806	0.01		SFGdor.R	7.612	2.69E-13
	SFGdor.L	-3.32	0.002	16	MFG.R	8.742	5.85E-17
6	MFG.L	-2.802	0.014		SFGdor.L	4.577	2.61E-05

7	MFG.L	4.296	1.29E-04		SFGdor.R	9.739	1.51E-20
	SFGdor.L	3.352	0.003	17	IPL.L	-3.792	3.28E-04
8	IPL.L	-4.035	2.56E-04		MFG.R	4.375	3.15E-05
	SFGdor.R	-2.635	0.02	18	IPL.L	-2.839	0.012
9	IPL.L	3.995	4.11E-04		MFG.L	4.933	6.41E-06
	SFGdor.L	3.754	9.62E-04		SFGdor.L	3.433	0.002
10	IPL.L	-3.172	0.007	19	SFGdor.R	3.007	0.013
	IPL.R	7.868	2.43E-13	20	MFG.L	-11.83	5.34E-31
	MFG.R	2.39	0.049		MFG.R	-14.5	1.92E-45
11	MFG.R	7.11	3.42E-11		SFGdor.L	-68.23	0
	SFGdor.R	4.948	1.11E-05		SFGdor.R	-93.95	0
12	MFG.L	-5.55	5.50E-07	21	IPL.R	3.885	2.64E-04
	MFG.R	-4.926	1.23E-05		MFG.L	-4.797	6.94E-06
					MFG.R	4.554	2.01E-05
					SFGdor.L	-21.789	4.15E-98
					SFGdor.R	7.121	1.20E-11

Abbreviations: IPL: Parietal Inf lobule; MFG: middle frontal gyrus; SFGdor: dorsal superior frontal gyrus; R: right; L: Left.

Table S5. Brain regions that showed task-related activation for each individual in the planning block (non-expert older group). Only regions of interest with $p < 0.05$ are shown.

Participant	Anatomical Region	t-value	p-value (FDR-corrected)	Participant	Anatomical Region	t-value	p-value (FDR-corrected)
1	IPL.L	4.003	3.30E-04	13	IPL.L	-6.647	1.57E-10
	MFG.R	2.857	0.013		IPL.R	-8.842	2.49E-17
2	IPL.L	-4.04	9.84E-04		MFG.L	6.663	1.48E-10
3	MFG.L	-4.888	1.43E-05		MFG.R	3.744	3.67E-04
	MFG.R	-4.201	1.22E-04		SFGdor.R	5.344	3.00E-07
	SFGdor.L	-4.201	1.22E-04	14	IPL.L	2.801	0.009
	SFGdor.R	-4.027	2.30E-04		IPL.R	2.918	0.006
4	MFG.L	-5.972	2.00E-08		MFG.L	-1.914	0.078
	MFG.R	-2.455	0.028		SFGdor.L	-2.504	0.019
	SFGdor.L	-5.021	2.74E-06	15	IPL.L	4.378	4.65E-05
	SFGdor.R	-3.152	0.004		MFG.R	-3.081	0.005
5	MFG.L	4.23	2.98E-04	16	IPL.L	-3.582	0.004
	MFG.R	3.643	0.002		MFG.L	-3.388	0.006

	SFGdor.R	5.05	1.69E-05		SFGdor.R	-2.628	0.031
7	MFG.R	-3.466	0.002	17	IPL.L	-3.349	0.002
	SFGdor.L	-4.202	2.30E-04		MFG.L	-2.394	0.027
8	MFG.L	4.709	3.04E-05		SFGdor.L	-9.055	2.14E-18
	MFG.R	2.838	0.014		SFGdor.R	-7.301	1.48E-12
	SFGdor.R	3.386	0.003	18	IPL.L	14.66	4.98E-46
9	IPL.L	6.602	2.53E-09		MFG.L	5.508	1.02E-07
	SFGdor.L	-3.184	0.008		SFGdor.L	4.667	7.56E-06
10	IPL.L	2.37	0.04		SFGdor.R	5.935	8.93E-09
	MFG.L	7.529	8.86E-13	19	IPL.L	8.061	2.25E-14
	MFG.R	5.415	3.04E-07		SFGdor.L	3.538	8.87E-04
	SFGdor.L	7.37	2.32E-12		SFGdor.R	3.536	8.87E-04
	SFGdor.R	6.141	5.18E-09	20	IPL.L	-2.806	0.018
11	MFG.L	-4.217	1.30E-04		IPL.R	-3.373	0.004
	SFGdor.L	-3.748	6.50E-04		MFG.L	-8.005	7.56E-14
	SFGdor.R	-2.585	0.023		SFGdor.L	-2.431	0.046
				21	MFG.L	-2.649	0.024

Abbreviations: IPL: Parietal Inf lobule; MFG: middle frontal gyrus; SFGdor: dorsal superior frontal gyrus; R: right; L: Left.

Table S6. Brain regions that showed task-related activation for each individual in the calculation block (non-expert older group). Only regions of interest with p < 0.05 are shown.

Participant	Anatomical Region	t-value	p-value (FDR-corrected)	Participant	Anatomical Region	t-value	p-value (FDR-corrected)
1	IPL.L	3.241	0.003	13	IPL.L	-8.63	1.57E-16
	SFGdor.L	-2.994	0.006		IPL.R	-8.138	4.42E-15
2	IPL.L	-7.816	7.98E-13		MFG.L	5.221	5.11E-07
	MFG.L	-3.72	0.002		MFG.R	3.503	9.32E-04
3	IPL.L	-2.901	0.008		SFGdor.R	5.609	6.61E-08
	IPL.R	-2.726	0.013	15	IPL.R	-2.658	0.025
	MFG.L	-5.086	1.88E-06		MFG.L	-4.417	8.75E-05
	SFGdor.L	-2.479	0.024	16	IPL.L	-4.386	4.12E-05
	SFGdor.R	8.724	4.59E-17		IPL.R	-3.726	5.33E-04
4	MFG.L	-3.32	0.01		MFG.L	-6.352	2.58E-09
5	IPL.R	2.419	0.032		MFG.R	-4.675	1.22E-05
	MFG.L	7.139	3.06E-11		SFGdor.L	-2.931	0.007

	MFG.R	6.862	1.73E-10		SFGdor.R	-6.661	5.64E-10
	SFGdor.L	3.649	9.31E-04		IPL.L	-5.71	7.40E-08
	SFGdor.R	8.793	1.29E-16	17	SFGdor.L	-4.989	2.74E-06
7	SFGdor.L	-2.798	0.011		SFGdor.R	-2.72	0.014
	MFG.L	10.47	7.82E-24		IPL.L	8.34	3.71E-16
8	MFG.R	5.262	7.12E-07	18	MFG.R	-2.519	0.017
	SFGdor.R	5.782	5.24E-08		SFGdor.L	7.127	3.42E-12
9	IPL.L	10.99	1.15E-25		SFGdor.R	5.032	9.95E-07
	SFGdor.L	-3.241	0.003	19	IPL.L	7.062	2.39E-11
	MFG.L	4.3	6.68E-05		MFG.R	-3.826	3.31E-04
10	MFG.R	2.867	0.011		IPL.L	3.576	0.001
	SFGdor.L	5.681	9.16E-08		IPL.R	4.542	2.56E-05
	MFG.L	-4.033	2.33E-04	20	MFG.L	9.534	1.23E-19
11	SFGdor.L	-2.915	0.009		MFG.R	3.608	0.001
	SFGdor.R	-2.952	0.008		SFGdor.L	4.643	1.73E-05
12	MFG.R	-2.837	0.013		SFGdor.R	5.79	9.68E-08
	SFGdor.R	-2.926	0.01	21	MFG.L	-3.354	0.003

	MFG.R	-2.275	0.044
	SFGdor.R	-2.226	0.049

Abbreviations: IPL: Parietal Inf lobule; MFG: middle frontal gyrus; SFGdor: dorsal superior frontal gyrus; R: right; L: Left.
